# Robots down-under: the current climate of robotic-assisted surgery education in Australia

**DOI:** 10.1007/s11701-024-01994-8

**Published:** 2024-06-08

**Authors:** Aditya Sakalkale, Shriranshini Satheakeerthy, Justin M. C. Yeung, Fiona Reid

**Affiliations:** 1 General Surgery, Western Health, 176 Furlong Road, St Albans, Melbourne, VIC 3021 Australia; 2 Colorectal Surgery Unit, Western Health, Footscray, VIC Australia; 3 Academic Department of Surgery, University of Melbourne, Melbourne, VIC Australia

**Keywords:** Robotic Assisted Surgery, Education, Surgical Education and Training, Australia

## Abstract

Australia has seen a significant rise in the use of Robotic-Assisted Surgery (RAS), with general surgery being the fastest-growing field in this technology. The proportion of general surgical RAS has grown from 1% to 17% of all RAS between 2008 and 2023 in Australasia. As of May 2023, there were 162 robotic platforms in Australasia, with 26 of them in the public sector. As the cost of establishing RAS decreases over time, public hospital robotics systems are expected to become more accessible. Despite the increasing demand, many specialties, including general surgery, do not have an agreed RAS curriculum for trainees. It is imperative for Australia to develop its own curriculum akin to our overseas colleagues to match this growth.

Much like our North American colleagues, Australia has experienced exponential growth in Robotic-Assisted Surgery (RAS) since its introduction in 2003. Like laparoscopic surgery, RAS was originally met with scepticism. Critics dismissed Dr. Kurt Semm’s laparoscopic appendicectomy in 1981, yet laparoscopic surgery is now a cornerstone in minimally invasive surgery, and RAS is following suit [1]*.* General surgery surmounted Urology and Gynaecology as the fastest-growing speciality in RAS within Australasia. Between 2008 and 2023, the proportion of general surgical RAS grew from 1% of all RAS to 17%, with colorectal procedures driving the growth [2]. Between 2010 and 2019, 6,110 general surgical procedures were successfully conducted using robotic assistance [3]. It is clear RAS has carved its niche in Australasia; however, in a publicly funded hospital system, overall uptake is slow and the current surgical training is not reflective of this impressive growth.

Australia’s universal healthcare system, Medicare, is funded by tax revenue and a government levy, covers patient healthcare costs. For example, a Low Anterior Resection, which typically costs AUD $23,900 (USD $15,500), would be covered. The current estimated cost of establishing a *‘da Vinci Xi (Intuitive Surgical, Inc.)*’ system in Australia is AUD $3.9 million (USD $2.5 million) combined with an additional AUD $621,245 (USD $399,404) service fee for a 3-year contract, creating a significant barrier for Medicare-based hospitals. As of May 2023, there were 162 robotic platforms in Australasia, 26 of which remain in the public sector [2] highlighting a socio-economic disparity.

With the increase in access to robotics, a corresponding increase in robotic training and robotic assisting is required; with the exposure to robotics (ideally) earlier in every surgeon’s career. This increase in exposure should shift RAS training into the formalised Surgical Education and Training programmes (SET, much like current American residency programmes), instead of developing these skills during fellowship or early consultant years as it currently is.

Many surgical specialties, including general surgery, lack an agreed RAS curriculum for trainees despite the increasing growth and willingness of these trainees to learn RAS. This needs to change. A survey conducted on surgical trainees in the UK and Ireland revealed that although 73.4% were interested in developing robotic surgery skills, only 12% reported having received training opportunities [4]. There are also varying opinions within trainee groups regarding the significance of robotic training. Around 14% of senior surgical trainees (U.K. ST3-5 training levels) expressed concerns about robotic teaching in the formative stage of their training. They felt that a curriculum mainly based on robotics might harm their ability to perform larger and open resections, a key skill they believed to be essential for successfully performing emergency operations [4]. However, many from the surgical community believe that robotic skills, like laparoscopic skills, are readily transferrable and are important for juniors to develop. In a poll of surgical residents at Yale and the University of Toronto for example, 95% agreed that robotic simulation training improved laparoscopic skills and 92.5% of participants found robotic simulation skills useful in performing general surgical procedures [5].

In the United States, the American Urological Association has made RAS part of their core curriculum for residents [2]; this is currently absent within Australia. For those trainees seeking a formalised and validated course in robotic surgery, the Department of Defence and the Institute for Surgical Excellence also offer a programme called "Fundamentals of Robotic Surgery" [6]. However, this can only serve as an adjunct to regular on-site teaching within hospitals, as illustrated by a North American study where 64.2% of respondents stated that simulation training did not substitute for actual operating room experience [5].

Attempting to address this issue of training in Australia, a roadmap was proposed by the Royal Australasian College of Surgeons (RACS) in 2023. Self-funded, one–two-day training courses have been developed by the International Medical Robotics Academy (IMRA), in collaboration with RACS [7] for continued professional development. This initiative acknowledges the increasing importance of robotic-assisted surgery (RAS) and also highlights that current RAS training courses in Australia are mainly sponsored by industry, leading to a potential conflict of interest [2]. A Robot-Assisted Surgery Working Party (RASWP) convened on July 2022 to evaluate the status and prospective training validation of RAS in Australia and presented their findings to RACS in June 2023 [2]. One notable point in this report was that SET trainees interested in post-fellowship robotic positions may be considered less competitive due to the lack of robotic exposure they received during the SET programme [2].

A sequential ‘up-skilling’ approach (proficiency-based progression) may be the best way to adopt competency-based assessments in robotic surgery. Similar to the ten-stage process suggested by the RASWP [2], a Robotic Education programme developed in Sao Paulo has been implemented with emphasis on four key pillars; online modules aimed to familiarise the trainee with communication nomenclature and the different operating platforms, bedside assisting to consolidate online teaching and to assist with ‘arm-docking’ and problem-solving, simulation with both virtual reality and physical procedures on organic tissue such as suturing/closing of tissue planes, and finally, once these three pre-clinical stages are complete, the trainee moves on to the final pillar which includes at least ten proctored procedures with staged progression [8].

Two main themes emerge from this new proposed curriculum. The first is the introduction of a pre-clinical objective proforma used to identify machine errors, and teach trainees nomenclature for safe assisting in addition to establishing surgical proficiency in docking and undocking robotic arms. Once complete, the second stage is the completion of physical proctored assessments. Proficiency-based progression assessments are similarly employed in the Belgian Robotic Surgery Academy ORSI; however, this is delivered in specific operation-based workshops spanning several days rather than having continuous education and training [9].

A potential solution to bridge the enormous gap between pre-clinical education and proctored cases on patients may already lie in the published literature. Metrics on how to assess trainees have been suggested in the Foundations of Robotic Surgery (FRS) curriculum; assessing *“knot tying, continuous suturing, cutting, dissection, and vessel coagulation”* [6]. These skills can be assessed by way of the validated Global Evaluative Assessment of Robotic Skills (GEARS) tool, completed by trained specialists [10]. Avian models have been established as an appropriate medium for assessment [11], and whilst in its infancy, Virtual Reality (VR) training models have also been shown to improve technical skills. This could be offered as an alternative (see Fig. 1) [12]. A proposed structure of robotic education can be seen in Fig. 1.

**Fig. 1:**
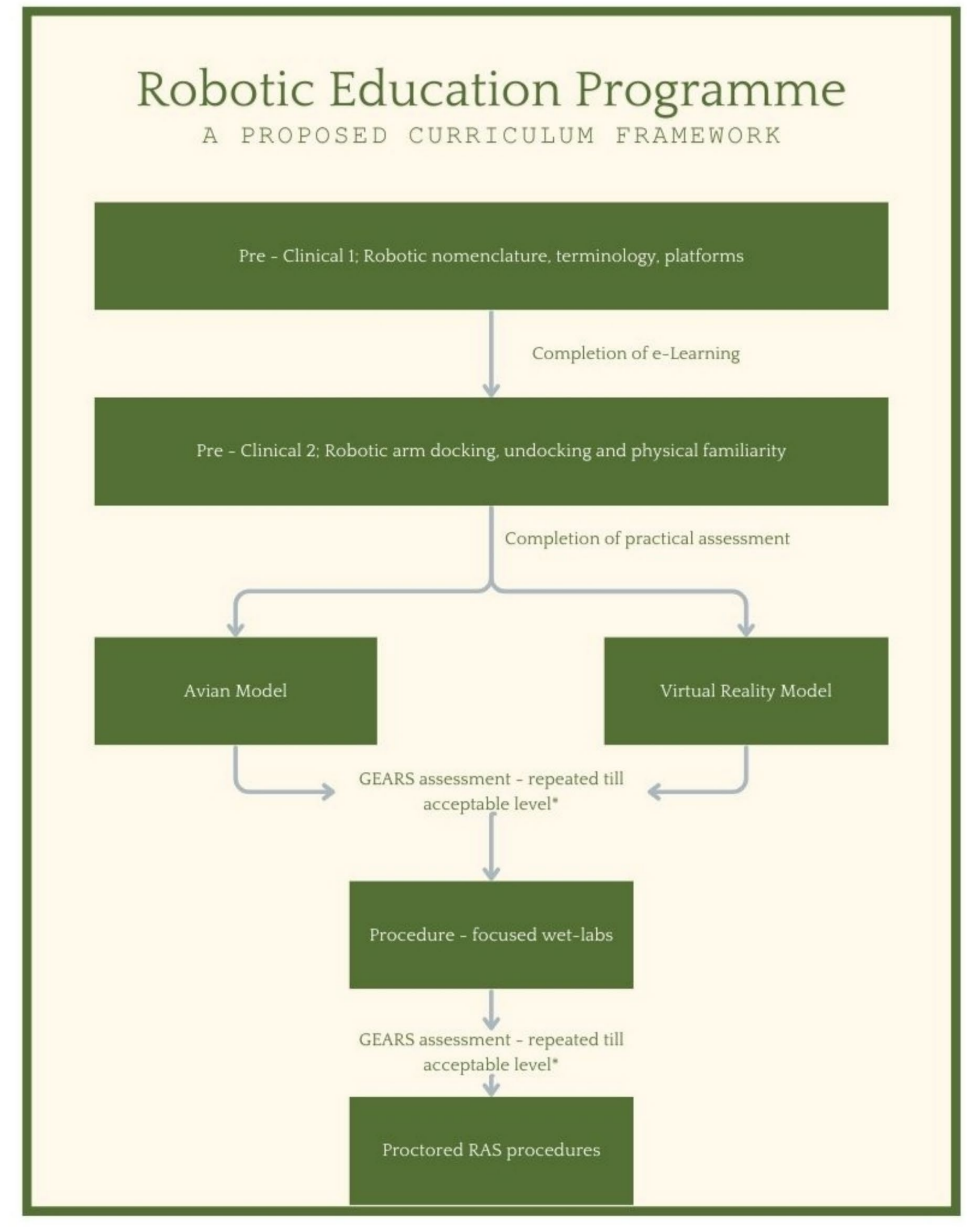
Robotic education programme: a proposed curriculum framework. The Avian Model/VR model to be used to consolidate ‘Foundations of Robotic Surgery’ assessable skills; *“knot tying, continuous suturing, cutting, dissection, and vessel coagulation”* * Acceptable levels are determined by specialists either at the hospital network level or by the overseeing training board

Surgical training in Australia must adapt to incorporate RAS as part of training, separate to that of open and laparoscopic training and ideally led by healthcare networks and regulated training bodies rather than industry. Simulation-based learning can be incorporated readily with minimal disruption to daily workflow. A core curriculum that encourages junior trainees to develop their skills, followed by a direct physical simulation laboratory component to consolidate learning will also need to be implemented. Now is the right time to invest in formal RAS training for trainee surgeons within the Australian SET curriculum.
